# Bioaerosols and Transmission, a Diverse and Growing Community of Practice

**DOI:** 10.3389/fpubh.2019.00023

**Published:** 2019-02-21

**Authors:** Samira Mubareka, Nicolas Groulx, Eric Savory, Todd Cutts, Steven Theriault, James A. Scott, Chad J. Roy, Nathalie Turgeon, Elizabeth Bryce, George Astrakianakis, Shelley Kirychuk, Matthieu Girard, Gary Kobinger, Chao Zhang, Caroline Duchaine

**Affiliations:** ^1^Department of Laboratory Medicine and Pathobiology, University of Toronto, Toronto, ON, Canada; ^2^Sunnybrook Research Institute, Toronto, ON, Canada; ^3^Department of Mechanical and Materials Engineering, University of Western Ontario, London, ON, Canada; ^4^Public Health Agency of Canada, Branch of Infectious Disease Prevention and Control, Applied Biosafety Research Program, Winnipeg, MB, Canada; ^5^Dalla Lana School of Public Health, University of Toronto, Toronto, ON, Canada; ^6^Department of Microbiology and Immunology, Tulane School of Medicine, Tulane University, New Orleans, LA, United States; ^7^Recherche de l'Institut Universitaire de Cardiologie et de Pneumologie de Québec, and Département de Biochimie, de Microbiologie et de Bio-Informatique, Faculté des Sciences et de Génie, Université Laval, Québec, QC, Canada; ^8^Department of Pathology and Laboratory Medicine, University of British Columbia, Vancouver Coastal Health Research Institute, Vancouver, BC, Canada; ^9^School of Population and Public Health, University of British Columbia, Vancouver, BC, Canada; ^10^Canadian Centre for Health and Safety in Agriculture, University of Saskatchewan, Saskatoon, SK, Canada; ^11^Institut de Recherche et de Développement en Agroenvironnement, Québec, QC, Canada; ^12^Centre de Recherche en Infectiologie, Université Laval, Québec, QC, Canada

**Keywords:** bioaerosols, microbes, virus, infections, viral dissemination, network, CANIBAN, collaborations

## Abstract

The transmission of infectious microbes via bioaerosols is of significant concern for both human and animal health. However, gaps in our understanding of respiratory pathogen transmission and methodological heterogeneity persist. New developments have enabled progress in this domain, and one of the major turning points has been the recognition that cross-disciplinary collaborations across spheres of human and animal health, microbiology, biophysics, engineering, aerobiology, infection control, public health, occupational health, and industrial hygiene are essential. Collaborative initiatives support advances in topics such as bioaerosol behavior, dispersion models, risk assessment, risk/exposure effects, and mitigation strategies in clinical, experimental, agricultural, and other field settings. There is a need to enhance the knowledge translation for researchers, stakeholders, and private partners to support a growing network of individuals and agencies to achieve common goals to mitigate inter- and intra-species pathogen transmission via bioaerosols.

## Modern Miasmas

The importance of infectious bioaerosols in disease transmission has been long-acknowledged, yet poorly understood. Paltry data and methodological heterogeneity limit many related studies. Effective ventilation and infection prevention and control (IP & C) measures in the form of droplet and airborne isolation in healthcare institutions underscore the contribution of these modes of pathogen dispersion. Moreover, recent outbreaks such as the Severe Acute Respiratory Syndrome coronavirus (SARS-CoV) and Middle East Respiratory Syndrome coronavirus (MERS-CoV) outbreaks highlight major gaps in our ability to assess and determine risk and to mitigate patient and healthcare worker (HCW) exposure alike (1, 2). The SARS outbreak was eventually controlled in the absence of an effective vaccine or antiviral, strictly through public health interventions and IP & C measures aimed at controlling, among other things, bioaerosols emitted by infected patients (3). A decade on from this experience, MERS-CoV has caused community and healthcare-associated severe acute respiratory infections in the Middle East and South Korea, spreading by similar mechanism(s) (4–6).

From an animal health perspective, pathogens such as porcine reproductive and respiratory syndrome virus may be transmitted through the air for significant distances and have significant economic impact on the agricultural sector (7). Efforts to characterize this and other relevant organisms have been undertaken previously, specifically looking at the burden of inoculum related to transmission (8) and the prevention of spread of aerosols through various adequate ventilation strategies (9–11). Emission of African swine fever virus into the air by infected pigs raises the possibility for droplet and/or airborne transmission of this virus, which has recently produced significant alarm after documented spread in China (12–14). This hemorrhagic virus is associated with high mortality and significant loss for producers through depopulation and trade restrictions (15, 16).

In recent years, new developments have enabled progress in bioaerosol research, thus establishing a community of practice in the field. Although many developments have been technical, one of the major catalysts has been the recognition that cross-disciplinary collaborations across the various spheres of human and animal health, microbiology, engineering, aerobiology, infection control, public health, occupational health, and industrial hygiene are essential. A network approach has proven successful in other cross-disciplinary fields, including One Health and eco-health whereby wildlife, computational and evolutionary biologists, microbiologists, virologists, epidemiologists, ecologists, environmental scientists, climatologists, and human, animal, and public health practitioners are collaborating to address challenges in zoonotic diseases research and control (17, 18). This has enhanced surveillance efforts and launched ambitious, large scale projects such as the Global Virome Project, though gaps remain in stakeholder engagement and monitoring activities (19, 20). Translation of bioaerosol research stands to benefit in a similar fashion, provided coordinated and committed efforts.

Potentially infectious bioaerosols are pertinent to a wide range of pathogens, some of which may be endemic and cause sporadic infections and outbreaks (e.g., *Mycobacterium tuberculosis* and *Legionella pneumophila*, the causative agents of tuberculosis and legionnaires' disease, respectively) and/or have potential to cause epidemics or pandemics (e.g., influenza virus A) (21–24). In addition, the importance of potentially infectious bioaerosols across different settings is underscored. These include, but are not limited to, agriculture (both crop and livestock), wastewater treatment plants, environmental reservoirs (soil and water), acute and long-term healthcare institutions, and shared public spaces (transportation hubs, recreational areas, etc.). Here, we discuss the state of the science for this nascent field and identify gaps requiring urgent attention.

## Value Proposition for the Study of Potentially Infectious Bioaerosols

Progress in the field has been stimulated by advances in other areas which have been applied to the study of infectious bioaerosols. These include: (a) enhanced detection by molecular methods, principally real-time and quantitative PCR, next-generation sequencing, metagenomics, and biosensors (25–30), and (b) establishment of both conventional and novel infrastructure, such as small and large-scale wind tunnels, biocontained rotating drums for aging aerosols, and field-ready aerosol samplers (31–33). Ongoing research has also facilitated the development and dissemination of procedures and protocols for experimental work, including artificial aerosols, as well as animal models of transmission including the ferret model for influenza virus transmission and macaque model for Ebola virus transmission (34, 35).

Whilst aerosols can be used as a means of delivery of therapeutic agents directly to the respiratory tract, they may also be used for the nefarious dispersion of human or animal pathogens. Scientists focused on bioaerosol generation, pathogen survival in air and aerobiological fitness must be acutely cognizant of any potential for dual use and routinely re-assess projects and proposed experiments with this perspective in mind. Oversight by institutional biosafety officers and committees may also consider this aspect of bioaerosol research during risk assessments and other internal approval processes.

Given the role of potentially infectious bioaerosols to transmit infectious agents to human and animal populations, it stands to reason that their detection and characterization will ultimately contribute to mitigating the spread of disease. This is possible through efforts focused on the following themes:

1. **Fundamental biological and physical properties of bioaerosols and their generation**
  Unresolved fundamental questions may be answered by studying infectious bioaerosols. Areas meriting attention are numerous and a few are briefly discussed here.
  In the case of agents for which airborne transmission is well-recognized (i.e., *M. tuberculosis*), associations between particle size and generation, pathogen content and virulence, as well as the deposition within the respiratory tract may be determined. The physical and chemical aspects of aerosols including the effects of relative humidity can affect pathogen viability and understanding these aspects of bioaerosol behavior may be beneficial to understanding conditions for controlling bioaerosol dispersion (36–38). Moreover, pathogens such as viruses may vary with respect to isoelectric points, possibly imparting different charges to infectious bioaerosols which may affect their behavior (39, 40). Finally, although climate change and pollution have been major issues within our biosphere, little is known about the impact of pollution particulates and gases, such as ozone, on the biology and physical properties of bioaerosols (41).
  Our understanding of bioaerosol production from expulsion events such as breathing, talking, sneezing, and coughing have generally been extrapolated from models, though more recent empiric evidence has become available, significantly enhancing our understanding of the transmission potential of bioaerosol emissions from naturally-infected hosts (42, 43). Awareness of factors contributing to particle velocity and penetration into space enables modeling strategies that inform engineering controls in a multitude of settings. For example, in healthcare, understanding the dispersion of potential pathogens in the environment can inform infection prevention and control practices (44). There is also potential benefit to determining pathogen characteristics. If enhanced infectious bioaerosol survival in air is associated with certain strains, genotypes or mutations, this provides possibilities for follow-on work to (a) determine the mechanism and (b) enhance surveillance. The latter would have implications for public and animal health. Studying these factors individually or in combination can optimize air handling and other mechanical, environmental or chemical means of neutralizing bioaerosols prior to host exposure, thus alleviating dependence on personal protective equipment, which is the last means of protection prior to exposure.
2. **Detection, surveillance, and early warning**
  New understanding of the role of bioaerosols in the propagation of human and animal pathogens could lead to new practices. As it stands, it remains uncertain which bioaerosol particles are predominantly responsible for person-to-person transmission. There may also be differences between pathogen populations in the infected host vs. what is emitted into the air. This poses an excellent opportunity to leverage advances in metagenomics with developments in aerobiology to extract more sophisticated data from aerosol samples and, potentially, determine genetic bottlenecks for transmission. This would feed back into more fundamental work on bacterial or viral determinants of transmission, as well as lead to novel means for surveillance and mitigation.
  Similarly, translational studies enhance our understanding of risk and determinants of exposure. For many settings and situations, several components of risk assessments are based on limited evidence. This is of particular concern from an occupational health perspective, in healthcare (e.g., purported aerosol-generating procedures), agricultural, or specific settings such as wastewater treatment plants (45–47). Developing the capacity to generate empiric data on pathogen content and biology from bioaerosols can lead to tools for outbreak investigation, surveillance, and development of risk assessment strategies and policies. As the technology for biosensors accelerates, the possibilities for rapid point-of-care testing and even remote sampling are highlighted. Integration of biosensors with bioaerosol sampling (26) has significant potential for early warning and public safety.
3. **Research and development of mitigation strategies**
  As advances are made, capacity is built to optimize and evaluate known and novel means to control the dispersion of infectious bioaerosols. These include the use of germicidal and pulsed ultraviolet light, mechanical air filtration and respiratory protection which have both mechanical and electrostatic filtering systems (48, 49). In addition, the potential to use ozone to control indoor bioaerosols in urban and rural settings is being examined. Ozone is a strong oxidizing agent having high redox potential and a short half-life, dependent on temperature. It has been used extensively in the food industry (specifically water treatment, washing of produce, and food preparation), as well as in domestic restoration to remediate odors and smoke damage. Its historical use and proven efficacy demonstrate its potential to remediate contaminated indoor air (50, 51). This would present a novel application of a known treatment modality.

## Gaps and Challenges

A number of important gaps have been identified (52) and these can be prioritized based on potential benefit:

### Infrastructure

Biocontainment of infectious particles is the first consideration when aerosolizing human and/or animal pathogens in an experimental setting. These systems are generally custom-designed and constructed, with few facilities capable of conducting aerosol work with risk group 2 pathogens, and fewer still with the capacity to work with risk group 3 or 4 agents.

### Technical

As challenges in infrastructure are overcome, opportunities to optimize and improve methods and techniques in bioaerosol research must be taken. A limitation of many studies focused on viral bioaerosols is the pervasive use of nucleic acid detection, rather than infectious virus isolation. The latter is a much more accurate indicator of the infectious potential of a bioaerosol but is infrequently performed due to poor sensitivity and other technical issues (53). The development of sampling devices and techniques optimized to preserve pathogen viability would considerably advance the utility of studying bioaerosols for risk assessment and management. For other applications, a more rapid, field-ready point of care test would be useful and would offer remote sampling possibilities. Biosensors have the potential to fill this gap and integration into aerosol sampling devices is under development (26). Finally, the collection of nucleic acid may be leveraged to obtain more sophisticated information than is available by PCR and Sanger sequencing. Metagenomics on environmental samples has been well-described in other spheres and is currently being explored for air samples (54–57).

### Standardization

To advance bioaerosol-related data interpretation, an effort must be made to standardize and share protocols and reagents to reduce the degree of data heterogeneity to enable meaningful analyses across studies. This may apply to animal models, artificial aerosolizations, collection strategies in the field and clinical settings, processing of samples, and detection methods. Developing or adopting a standardized approach to aerosol sampling is challenging and requires substantial efforts to select the best sampling strategy to minimize sampling biases, optimize sample concentration and organism retrieval whilst preserving integrity (58).

**Figure 1:**
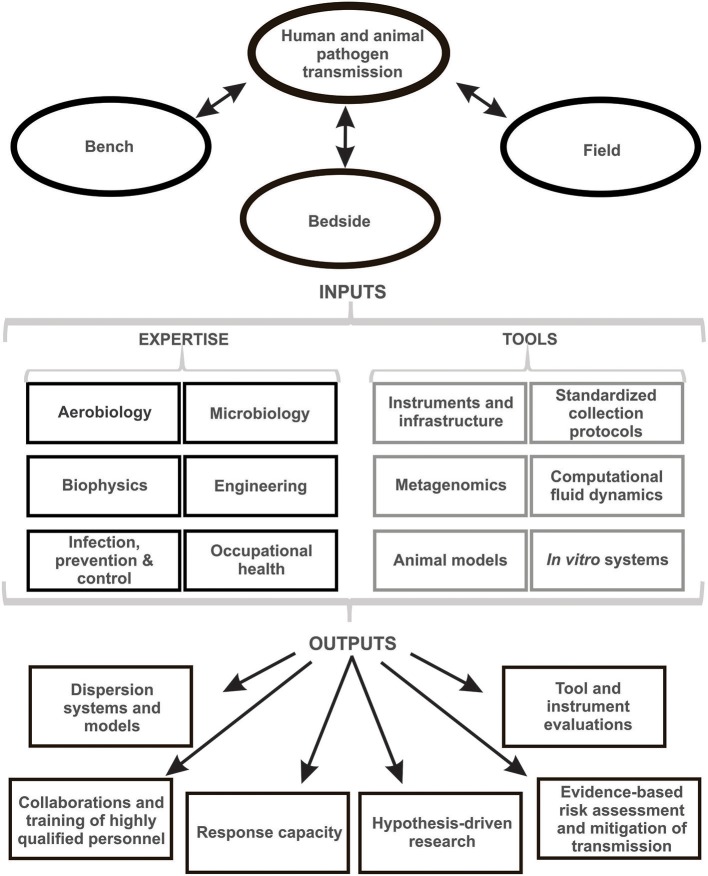
Summary of the research fields, assets, and deliverables for collaborative bioaerosol research.

Establishing standard approaches also enables training and implementation and eases knowledge translation amongst different groups (Figure 1). Multiple investigators have published small studies on the recovery of viral RNA emitted by naturally infected humans using different approaches for recruitment, sampling, processing, and detection. Unfortunately, the results are difficult to compare and, standing alone, are of limited statistical significance (45, 59–61). By standardizing approaches, a more feasible method can be used to compare separate studies allowing for larger multi-center studies that are substantially more conclusive and impactful.

### Training

Additional educational and operational needs, such as training of research personnel, proper use of personal protective equipment and developing decontamination protocols must be addressed. As more highly qualified personnel are properly trained, the greater the capacity for designing and utilizing experimental systems and for completing meaningful clinical and field studies. The need for cross-disciplinary experience is underscored, since robust knowledge of physics, mechanical engineering, and microbiology are required. Fostering this expertise requires a collaborative effort as each discipline offers unique insight. Currently, there are a limited number of workshops or accredited courses available to cultivate both interest and baseline knowledge. Although the profile of bioaerosol research is rising, few trainees are exposed to the field early in their careers. Courses such as *Bioaérosols et aérobiologie* (Bioaerosols and Aerobiology) developed at the Université Laval in Québec may help close this gap further given the opportunity to expand reach.

### Knowledge Translation and Response

The relevance of bioaerosol data to occupational health and infection prevention and control requires attention. Validation of bioaerosol data for risk assessments and risk management is needed. Air sampling was conducted during both SARS-CoV and MERS-CoV outbreaks, accruing important insight into the environmental distribution and persistence of these coronaviruses (4, 62, 63). The absence of data for more common, lower consequence pathogens represents missed opportunities to build a contextual and impactful body of knowledge during inter-outbreak and inter-pandemic periods.

To develop evidence-based policy and ensure the relevance of this work, early stakeholder engagement is needed. Consultation with human and animal public health, infection prevention and control, industrial hygiene, and occupational health stakeholders will ensure that the work is germane to the challenges presented by bioaerosol exposures. This pull also increases the likelihood that there will be data generated for risk assessment, policy development and implementation.

## The Road Ahead; Solutions and Recommendations

Part of the chaos that characterized the SARS epidemic can be attributed to a lack of knowledge regarding the route(s) of transmission. This remains true for high consequence coronaviruses, as well as for a range of other respiratory pathogens, both novel and established. We propose the following to address the challenges outlined above and to further develop the field of applied bioaerosol research:

1. **An open network approach**
  Working in isolation, microbiologists, aerobiologists, engineers, and epidemiologists have made only incremental progress. A synergistic and pluralistic approach is required for action-driven research which effects change. As capacity grows, so will opportunities for training and response. A networked approach will lead to context-specific follow-on research to ultimately inform policy for the mitigation of respiratory pathogens spread in healthcare institutions, agricultural settings and public spaces. Toward this end, the present authors have established the Canadian Infectious Bioaerosols Network (CANIBAN) that brings together members of each of these disciplines with the primary objectives of understanding transmission of pathogens.
2. **Shared infrastructure, technical protocols, and training programs**
  A network would form a hub around which key resources such as infrastructure, equipment, and operational procedures could be shared, thus also enabling training and underscoring best practices in biosafety and biosecurity. Shared resources may encompass wind tunnels, cough chambers and mannequins, animal exposure and other biosafety enclosures, and rotating drums to examine pathogen survival in air, together with instrumentation and analysis tools for fluid flow measurement and biochemical assays.
3. **Identifying and engaging knowledge-users**
  Limited awareness and understanding of infectious bioaerosols among potential knowledge users, coupled with equally limited outreach by bioaerosol researchers has restricted knowledge transfer and applications. Knowledge-user engagement in research planning from the outset and ongoing involvement through to dissemination is key for effective projects. Potential knowledge users include infection prevention and control practitioners, infectious disease specialists, veterinarians and animal health epidemiologists, industrial hygienists, and public health agencies.
4. **Enhanced capacity-building for response measures** Increasingly, bioaerosol sampling has been proposed and implemented in outbreak investigations and pathogen surveillance using *ad hoc* approaches. The development of best practices in these areas is essential to generating valid and actionable data.

In summary, a collective path for researchers, stakeholders, and private partners is needed to support a network of individuals and agencies to achieve common goals. As the field of bioaerosol studies grows, applications will diversify, along with novel technologies for remote sampling and point of care testing yielding results in real time. This stands to mitigate the spread of nefarious pathogens and contribute to early warning and response measures, thus ultimately benefiting human and animal health.

## Author Contributions

SM framed the manuscript and contributed content. NG contributed content and generated the figure. ES contributed content and supported manuscript organization. TC, ST, JS, CR, NT, EB, GA, SK, MG, GK, and CZ contributed content and expertise and CD helped to frame and contributed content.

### Conflict of Interest Statement

The authors declare that the research was conducted in the absence of any commercial or financial relationships that could be construed as a potential conflict of interest.
